# 

**Published:** 1864-01

**Authors:** 


### INDEX TO VOLUME XVIII.

A Peculiar Case. By A. S. Talbert, D. D. S. ---------- 14
A Quack Recipe ---------- 32
Alleged Death from Inhalation of Nitrous Oxyde Gas ---------- 48
A Case ---------- 5
An Address ----------55, 63, 202, 509
Anæsthesia ----------87, 124
A Chloroform Case ----------98, 238
A Medal-some Association, ---------- 100
An Address ---------- 105
Automatic Plugger ----------148, 191
A Strange Case, ---------- 180
Academy of Sciences,—Prizes, ---------- 181
Another Medal, ---------- 193
A New Application of Electricity, ---------- 219
American Dental Association, ----------240, 424
A Trip East, ---------- 281
A Reply to the Editor and Publisher of the Dental Cosmos, ---------- 335
A New Method of Grafting Crowns upon the Roots of Teeth, ---------- 347
A Wheel within a Wheel, ---------- 371
Address of Dr. P. G. Hunt, ---------- 372
American Dental Convention, ----------423, 453, 485
Alveolar Dental Periostitis, ---------- 427
A Case, ---------- 507
Aggregate Labor of Mankind, ---------- 547

Brooklyn Dental Association, ----------309, 398
Bromide of Potassium in Inducing Sleep, ---------- 416
Brooklyn Dental Association, ---------- 540

Correspondence, ----------22, 85, 115, 267, 371, 400, 468
Central New York Dental Association, ---------- 21
Chicago Dental Society Organization ---------- 175
Conveyance of Sound by the Teeth, ---------- 178
Cheap Food in Paris, ---------- 182
Case of Traumatic Tetanus, ---------- 235

Connecticut Valley Dental Association, 258
Chicago, 270
Close of the Volume, 556

Directions for using the Plastic Metallic Filling, 9
Dental Education, 149
Dental Periostitis, 195
Dental Hygiene, 206
Dental Association, 237
Diseases of the Maxillary Sinus, 287
Dental Fogyism, 297
Dental Pupilage, 441
Death from Chloroform, 510
Dental Polychrists 49
Duties and Responsibilities of the Dental Profession, 519
Delay 555

Encephaloid Cancer of the Left Superior Sinus, 30
Editorial, 5
Electricity as the Principle which Causes the Vitality and Coagulating Property of the Blood, 183
Enlighten the People, 630
Effects of the Excessive Use of Sugar on the System, 549
Filling Teeth, 152
Filling Pulp Cavities and Canals, 331

Gold Palate Rubber Work, 444
Going to Paris, 147
Growth of Bone 279
Gone to the War, 285
Gold Foil, 334
Gold Webb and Rubber Combination, 515
Grafting Animals, 548

How to Enjoy the Toothache, 182
How a Clergymen Cured his Appetite for Tobacco, 548

Investigations Touching the Use of Iodine, 31
Inflammation, 61
Iowa State Dental Association, 74
Influence of the Times upon the Dental Profession, 192
Influence of Example, 293
Information for the People, 301
Instruments, 334
Indiana State Dental Association, 361
Iowa Dental Society 446

Lodgment of False Teeth in the Œsophagus 33
Laughing Gas, 41, 139
Letter from Phil, 115
Literature in Medicine, 221

Meetings of Societies, 5
Michigan Dental Association, 66
Mississippi Valley Association, 99
Materials of which the Sun is Made, 137
Mississippi Valley Association of Dental Surgeons, 159
Meeting, 425
Merrimack Valley Dental Association, 506

Northern Ohio Dental Association, 260
Nitrate of Silver, 28
Nitrous Oxide an Anæsthetic, 146
New Dental Societies, 194
Necrosis of the Right Inferior Maxillany Bone, 198
New Haven Dental Society, 329
Nitrous Oxide Gas an Anæsthetic, 351, 402
New Teeth, 425
Notice, 542
Nerve Extractors, 555

On the Therapeutic Properties of Carbolic Acid, 25
On the Disposition made of Communications, 108
On Anæsthesia, 211
On the Changes of Form of Mammalian Blood-Corpuscles, 277
Obituary, 286
"O. U. C." H., 400
Ohio College of Dental Surgeons, 426
Ohio Medical College, 430
Observations on "Disease." 543
On the Application of Dialysis in Determining the Nature of the Crystaline Constituents of Plants, 550
On the Purity of Foreign Iodide of Potassium, 551

Plasticity of Blood Corpuscles, 43
Plaster Filling, 85
Professional Elevation, 101
Prize Essay on Anæsthetics, 193
Practical Medicine, 235
Professional Etiquette, 241
Penn Association of Dental Surgeons, 261, 354
Prolongation of Anæsthesia by Chloroform, 279
Polishing Powders, 285

Present Condition of an Old Case, 285
Physiological Influences and Hygienic Uses of Protoxide of Nitrogen, 365
Personal, 380
Physician's Visiting List and Book of Engagements, 425
Professional Fees, 434
Professional Egotism, 478
Peridental Inflammation, 500
Popular Essays by the Michigan Dental Association, 517
Preservation of Chloroform, 551

Relative Claims of Operative and Mechanical Dentistry, 227
Reminiscence—Valedictory, 552

Semi-Annual Call of the Western Dental Association, 237
Suggestions to the Dentist's Assistant, 239
Six Year Molars, 215
Society Notice, 280
Success, 377
Successful Treatment of Aggravated Diseases, 379
Specialties in Dentistry, 478
Steam Pressure in Vulcanizers, 527
Still at the Old Stand, 555

The Rubber Patent Question Again, 512
The People's Dental Journal, 240, 516
The Dentist's Memorandum and Engagement Book for 1865, 516
The Silver Medal, 468
The Dentist's Memorandum and Engagement Book, 472
The Dental Luminary, 472
The Demands of the Age upon our Profession, 381
The Dentist's Duty to his Profession, 383
The Causes of Exudation in Inflammation, 405
The Schools of Boston, 418
Treatment of Six Year Molars, 348
The Efforts of the Dental Cosmos to Render Justice, &c. 305
Transactions of the Odontographic Society, 330, 424
The Annual Meetings, 333
Tempering Instruments for Cutting, 255
Toothache—Inflammation of the Antrum, 257
The Vermont School Journal, 223
Teaching, 177
The Coagulation of the Blood, 187
The Good Physician, 188
The Terchloride of Carbon, 190

The Ohio Dental College Association, 119
The Effects of Congelation upon Water 143
To Improve the Solubility of India Rubber, 96
The Preservation of Natural Teeth, 15
The New (or Old) Anæsthetic, 36
The Eighth Annual Meeting of the Western Dental Society, 532
The Brooklyn Dental Association, 537

Unbecoming a Gentleman, 181

Wood's Alloy 192
Western Dental Society, 315
Wabash Valley Association, 380, 481
Why are not our Dental Schools better sustained by the Profession, 469
What is an Organism, 272
What Form of Mercury is contained in Rubber as a Base for Artificial Teeth, 554

Ventilation of Sleeping Apartments, 364

### DENTAL REGISTER

### OF THE WEST,

ISSUED MONTHLY, AT $3 A YEAR IN ADVANCE.

DEVOTED TO THE INTERESTS OF THE DENTAL PROFESSION.

EDITED BY J. TAFT AND GEO. WATT.

The Volume commences in January and closes in December.

The Register being issued monthly is always fresh, therefore indispensable to the practitioner who desires to keep posted up with the new inventions and improvements which are daily developed. Neither expense nor effort will be spared to give value and variety to its contents. Articles will be illustrated with well executed engravings. whenever they will add to the interest or assist in explanation.

Its extensive circulation and frequency of issue makes it one of the BEST DENTAL ADVERTISING MEDIUMS in the United States.

RATES OF ADVERTISING.

1 page, 1 year, $50. ½ page, 1 year, $30. ¼ page, or card, 1 year, $20.

All advertising bills payable in advance, or at furthest after the issue of the first number containing the advertisement. All transient advertisements to be paid for in advance.

☞ CONTRIBUTIONS SOLICITED.

Address Original Communications to **Geo. Watt, Xenia, o.**

Exchanges to **J. Taft, or Dental Register, Cincinnati, Ohio.**

Business Communications to

J. TAFT, Publisher,
172 Race Street.

